# A needs assessment for self-management services for adults awaiting community-based mental health services

**DOI:** 10.1186/s12889-023-15382-8

**Published:** 2023-03-27

**Authors:** Cally Jennings, Ben Singh, Helen Oni, Anna Mazzacano, Carol Maher

**Affiliations:** 1 Sonder Mental Health Services, Adelaide, South Australia Australia; 2 Alliance for Research in Exercise Nutrition and Activity (ARENA), University of South Australia, GPO Box 2471, Adelaide, South Australia SA 5001 Australia

**Keywords:** Mental health, Needs assessment, Lifestyle, Health services, Anxiety, Depression

## Abstract

**Background:**

High demand for services has resulted in lengthy waiting times being experienced across mental health services, both across Australia and internationally. Timely access to services is necessary to optimise the effectiveness of treatment, and prevent further mental health decline, risk of suicidality and hospitalisation for clients waiting for services to commence. The present study aims to better understand the experiences of individuals who are waiting for ongoing mental health services to commence and their preferences for additional support whilst on the waitlist, as a means to recommend alternative supports.

**Methods:**

A link to the cross-sectional, anonymous survey was sent via text message to 2,147 clients of a mental health service, with a reminder text message sent approximately one week subsequent to those who did not opt out of the communication. Eligibility criteria included having been a client of the service in the previous 12 months, having spent time on the waiting list, being aged 16 or over and having sufficient English proficiency.

**Results:**

A total of 334 participants responded to the needs assessment survey, 277 (82.9%) of which resided in the metropolitan region and 57 (17.1%) residing in the country region. Of the respondents, the majority presented with generalised anxiety/panic attacks (*n* = 205, 61.4%), followed by life stressors (e.g., financial concerns, relationships, *n* = 196, 58.7%) and lack of motivation/loss of interest (*n* = 196, 58.7%). Most respondents (52.7%) waited 4–12 months for ongoing services to commence and almost half (47%) reported that their mental health deteriorated during this time. Of the additional support options, most participants expressed interest in additional mental health supports (78.4%, *n* = 262), such as telephone support and access to online materials. There was significant interest in other supports such as exercise support (57.4%, *n* = 192), sleep education (56.6%, *n* = 190) and healthy eating support (41%, *n* = 137).

**Conclusion:**

Mental health services are experiencing significant waiting times, increasing the risk of mental health deterioration for persons waiting for services to commence. However, the findings demonstrate that there is interest for alternative support options, such as lifestyle interventions, in the interim. Desire for lifestyle support services, particularly in-person exercise programs and self-directed sleep, was especially high amongst the population of respondents within this study. Future work to rigorously develop and evaluate such lifestyle support services for mental health clients is warranted.

**Supplementary Information:**

The online version contains supplementary material available at 10.1186/s12889-023-15382-8.

## Background

The prevalence of mental health concerns is a growing concern both nationally and internationally, with one in eight people experiencing mental health conditions [1]. Within Australia specifically, the latest Australian Bureau of Statistic (ABS) data [2] demonstrates that at least two in five (43.7%) Australians aged 16–85 years experienced a mental or behavioural condition in their lifetime, and one in five experienced mental ill-health during 2020–2021 [2]. This was a significant increase of 22.6 percentage points from the 2017–2018 reporting period [3]. Individuals between the ages of 16–24 years (36.6%) had the highest rate of reported mental health conditions, followed by people aged 24–34 years (27.1%) in 2020–21 [2]. According to the Australian Institute of Health and Welfare (AIHW), mental and substance use disorders contributed to 13% of Australia’s total burden of disease in 2018, making it the second highest disease group contributing to non-fatal burden [3]. Certain population subgroups are at higher risk of mental health concerns due to greater exposure and vulnerability to unfavourable social, economic, and environmental circumstances, interrelated with gender [4]. For instance, statistics demonstrate that 57.2% of persons who identify as Lesbian, gay, bisexual, transgender, queer and intersex (LGBTQI +) and are aged 18 and over reported high or very high levels of psychological distress, compared to 15% in the general population [5]. Similarly, mental health concerns such as anxiety, depression, and drug use was the leading cause of non-fatal burden of disease amongst Indigenous Australians in 2018 [6].

While government expenditure on mental health support services continues to increase, many people experiencing mental illness do not receive timely treatment and support due to compromised health service access [7, 8]. As a result, individuals awaiting access to mental health support services may experience preventable physical and mental deterioration, disruptions in education and employment, relationship breakdown, stigma, and loss of motivation and opportunities [9, 10]. Emphasis is placed on preventing and managing common mental health conditions such as anxiety and depression by ensuring timely treatment, as well as by supporting clients and communities to engage in interventions that promote psychological well-being and reduce poor mental health [4, 11, 12]. Therapeutic lifestyle habits include exercise, nutrition and diet, recreation activities and relaxation, are found to be as effective as either psychotherapy or pharmacotherapy and can offer significant therapeutic advantages for people with low to moderate severity mental ill-health conditions [11–13].

While evidence indicates that these lifestyle factors have a positive effect on psychological wellbeing, help to reduce severity and risk of depression and anxiety [14, 15]; these cost-effective and healthy self-management approaches are often underutilised and seldom incorporated in clinical practice guidelines in the management of people awaiting mental health services [8]. There is a critical need to incorporate lifestyle medicine, such as healthy eating, exercise and sleep, within mental health interventions; particularly for populations who are on waiting lists for mental health services.

Therefore, this study aims to better understand the experiences of clients who are waiting for services to commence within a primary mental health service and explores their interest in and preferences for additional support include lifestyle interventions whilst on the waitlist. We also aim to evaluate whether there are differences in preferences based on gender, geographical location, Aboriginal and/or Torres Strait Islander status, and LGBTQI + status, to identify the need for targeted interventions. Findings will be used to inform the development of low-intensity lifestyle programs that may support clients while on the mental health service waiting list.

## Methods

### Overview of sonder

Sonder is a not-for-profit primary health care organisation that provides health services throughout metropolitan, regional and rural regions of South Australia. Approximately 70% of Sonder services provide support to individuals experiencing poor mental health, including mental health counselling, drug and alcohol treatment, employment support and Aboriginal health services. Individuals referred to the service are triaged and put on a waiting list of up to six months for face-to-face services, primarily due to the increasing demand of mental health services experienced across Australia relative to the number of clinicians. Whilst the organisation offers alternative services and support for clients who are waiting for ongoing services to commence, such as check-in calls and a Walk-in Service, some clients may experience deterioration of their mental health and physical health during this time. Providing low-intensive lifestyle interventions to individuals on the waitlist could potentially promote better mental health outcomes and facilitate increased support throughout the entire client journey.

### Design

This needs assessment used an anonymous, cross-sectional, online survey to understand interest in, and preferences for (modality, frequency and delivery approach) various types of low-intensity lifestyle supports among people waiting to access community mental health services. The project was approved by the University of South Australia's Human Research Ethics Committee (protocol number 204329). Potential participants were provided with a comprehensive information sheet, as well as information about the study on the first screen of the survey platform prior to commencing the survey. Persons contacted were able to opt out from communications or opt in to complete the survey by selecting the link, and completion of the survey was deemed to indicate informed consent. This project was conducted in accordance with the Declaration of Helsinki. Written informed consent was obtained from all participants.

### Participants

Eligibility criteria for participation included being a previous client of the organisation in which the study took place, persons who had previously spent time on a waiting list before receiving mental health services, aged 16 years or older, and being able to speak English at a sufficient level to complete the questionnaire. To ensure accuracy of responses, the survey was limited to clients who had accessed the service in the previous 12 months.

### Measures

The questionnaire was developed from a review of the literature undertaken through a critically appraised topic (CAT) which explored the effectiveness of different types of low-intensity lifestyle interventions for improving mental health. The results of the CAT informed identified lifestyle interventions with a sufficient evidence base to form the basis of survey questions (e.g., exercise, healthy eating and sleep). A working group was then formed consisting of representatives from the research team and Sonder staff to co-developed and finalise the survey. Staff included in the working group were the Mental Health and Alcohol and Other Drug Project Officer, Health Promotion Manager, Performance and Innovation Coordinator, Research and Evaluation Coordinator and Research and Evaluation Officer. In addition to the working group, the Clinical Leadership Group, consisting of the Mental Health and Alcohol and Other Drug Manager and Clinical Leads across the Mental Health and Alcohol and Other Drug portfolio were asked to review and provide feedback to assist in the development of the survey. This was done through regular updates at their meetings, by the Mental Health and Other Drug Project Officer and feedback of comments to the working group.

The final survey developed consisted of 22 questions pertaining to client demographics, experience whilst on the waitlist, preferences for different low-intensity lifestyle support and modes of delivery in which participants would have been interested in accessing while on the waitlist for ongoing mental health services.

Demographic questions included age, gender identity [16] (options included male, female, and non-binary, prefer not to answer, and a different identity, which included an open-text box), Aboriginal and/or Torres Strait Islander origin, ethnicity and whether they identified as LGBTQI + . Further demographic questions pertained to cultural and linguistically diverse background, country of birth and primary language spoken at home. Questions were also included to understand participants’ primary mental health concern for which they were referred for, length of time participants were on the waitlist, change in mental health while waiting for services and if they engaged in any activities while on the waitlist to improve their mental health (e.g. walk-in services, mobile phone apps such as smiling mind, lifestyle changes).

Lifestyle topics included exercise, healthy eating, sleep and additional mental health supports. The initial question for each lifestyle topic was framed to determine interest in the lifestyle topic being asked about. Response options were provided on a 5-point Likert scale ranging from ‘Very Interested’ to ‘Very Uninterested’. Answer options for ‘Very interested’ and ‘Somewhat interested’ were combined to provide an indicator for interest in the lifestyle topic. Following each initial question, additional questions asked participants about their preferred format, modality and timing of each lifestyle program. Answer options are further outlined in Table 3.

### Procedure

The retrospective study was conducted in South Australia and included participants who were based in both Country and Metropolitan regions. Potential participants were screened for eligibility through an in-house client management system. Eligible participants were sent a text message inviting them to participate in the study. The text message contained a link to complete the survey electronically via Survey Monkey, as well as a link to the Study Information Sheet. A reminder text message was sent approximately one week following the original message.

The survey was distributed to 2,147 clients from 10 to 23 March 2022, with reminders sent to 1,867 clients who had not opted out from receiving the reminder. To encourage participation, the survey included an optional random prize draw with the prize being a $250 Coles gift voucher. Participants who completed the survey and who agreed to provide their contact details for the purpose of the prize draw were entered into the draw.

### Analysis

Demographic characteristics were reported as means (SD) or n (%), and survey responses were reported as n (%) for the entire sample. Subgroup analyses for gender, geographical location, Aboriginal and/or Torres Strait Islander status, and LGBTQI + status were undertaken using Chi square tests. Holm-Bonferroni adjustments for multiple testing applied to all subgroup analyses to adjust for multiple comparisons. There were 12 comparisons for exercise preference, 9 comparisons for exercise type, 14 comparisons for healthy eating preference, 12 comparisons for sleep education preference, and 12 comparisons for other mental support interest, for each subgroup (gender, geographical location, Aboriginal and/or Torres Strait Islander status, and LGBTQI + status). Effect sizes for Chi square tests were calculated using Phi (*φ*), with effect sizes of 0.1 considered a small effect, 0.3 a moderate effect, and > 0.5 a large effect. *P* values < 0.05 were considered statistically significant.

Given that the study’s aims are descriptive in nature, a target sample size was not strictly pursued. Rather, attempts were made to maximise the study’s response rate and resulting sample. A priori power calculations for the subgroup analyses suggested that a total sample of *n* = 88 would provide 80% power to detect a moderate effect size difference (*φ* = 0.3) with an alpha of 0.05 for two group comparisons (e.g., male vs female, metro vs regional, Aboriginal status and LGBTQI + status) while a total sample of *n* = 785 would be required to detect an small effect size difference (*φ* = 0.1).

## Results

### Overview of sample

A total of 334 participants completed the survey, representing a response rate of 15.6%. Demographic characteristics of the sample are shown in Table 1. Mean participant age was 41.7 (SD = 15.4) and most were female (*n* = 235, 70.4%) and from metropolitan/urban geographical locations (*n* = 277, 82.9%). Participants were predominantly born in Australia (*n* = 266, 79.6%). Fourteen percent identified as LGBTQI + (*n* = 46) and 4.3% (*n* = 14) as Aboriginal and/or Torres Strait Islander.

**Table 1:** Demographic characteristics of the entire sample (*n* = 334)

	Total sample Mean (SD) or n (%) *n* = 334
Age, mean (SD)	41.74 (15.4)
Gender	
Female	235 (70.4%)
Male	63 (18.9%)
Non-binary	6 (1.8%)
Other	1 (0.3%)
Missing or prefer not to say	29 (8.7%)
Geographical location	
Country/rural	57 (17.1%)
Metropolitan/urban	277 (82.9%)
Aboriginal and/or Torres Strait Islander	
Yes	14 (4.3%)
No	292 (87.4%)
Missing or prefer not to say	28 (8.4%)
Culturally and Linguistically Diverse Background	
Yes	37 (11.1%)
No	270 (80.8%)
Missing or prefer not to say	27 (8.1%)
County of birth	
Australia	266 (79.6%)
Other	37 (11.1%)
Missing or prefer not to say	31 (9.3%)
What cultural background or ethnicity do you identify with?	
Australian	228 (68.3%)
Other	72 (21.6%)
Missing or prefer not to say	34 (10.2%)
Language spoken at home	
English	287 (85.9%)
Other	13 (3.9%)
Missing or prefer not to say	34 (10.2%)
Do you identify with LGBTIQ + ?	
Yes	46 (13.8%)
No	259 (77.5%)
Missing or prefer not to say	29 (8.7%)

### Use of services

An overview of the participants’ use of services is shown in Table 2. The most common mental health concerns at time of first referral to the use of services were panic attacks/generalised anxiety (*n* = 205, 61.4%), life stressors impacting on mental health (e.g., financial concerns, relationships, *n* = 196, 58.7%) and a lack of motivation/loss of interest in things they enjoy (*n* = 196, 58.7%). Just under half the participants were on the waiting list for less than four months (47.3%), with the remainder waiting four to 12 + months (52.7%). Around half of participants reported that their mental health deteriorated whilst on the waiting list (*n* = 156, 47%), whilst one third (35.5%) reported that it was stable.

**Table 2:** Overview of the use of services among all participants

	Total sample n (%)
What was your main mental health concern(s) when you were first referred to Sonder? (Tick all that apply)	
Panic attacks/generalised anxiety	205 (61.4%)
Lack of motivation/Loss of interest in things I enjoy	196 (58.7%)
Life stressors impacting on mental health (e.g., financial concerns, relationships)	196 (58.7%)
Persistent low mood/sadness	191 (57.2%)
Trouble sleeping (too much or too little)	183 (54.8%)
Excessive worrying	170 (50.9%)
Coping with traumatic events	136 (40.7%)
Social withdrawal/isolation	136 (40.7%)
Suicidal thoughts or self-harming	122 (36.5%)
Anger/irritability; frequent emotional outbursts	120 (35.9%)
Frequent mood swings	106 (31.7%)
Chronic pain impacting on mental health	78 (23.4%)
Grief and loss	72 (21.6%)
Other	35 (10.5%)
Substance use	26 (7.8%)
How long were you on the waiting list for?	
Less than 1 month	46 (13.8%)
1–3 months	112 (33.5%)
4–6 months	99 (29.6%)
7–9 months	38 (11.4%)
10–12 months	19 (5.7%)
Longer than 12 months	19 (5.7%)
Missing	1 (0.3%)
How did your mental health change while you were on the waiting list?	
Got a lot better	16 (4.8%)
Got a bit better	40 (12.0%)
Did not change	118 (35.3%)
Got a bit worse	89 (26.6%)
Got a lot worse	68 (20.4%)
Missing	3 (0.9%)
Was there anything you did while on the waiting list to try and improve your health? (Tick all that apply)	
Lifestyle Changes (e.g., exercise, healthy eating, improved sleeping)	125 (37.4%)
I did not try anything while on the waiting list	88 (26.3%)
Other	81 (24.3%)
Telephone Services (e.g., Beyond Blue)	57 (17.1%)
Mobile Phone Apps (e.g., Smiling Mind)	59 (17.7%)
Sonder Walk-in Service	27 (8.1%)
Online Treatment Programs (e.g., myCompass, MindSpot)	20 (6.0%)

### Interest in additional services

An overview of participants' interests in the use of additional programs is shown in Table 3.

**Table 3:** Overview of the interest in other services among all participants

While on the waiting list, how interested would you have been to be supported to do an exercise program?	
Very Interested	93 (27.8%)
Somewhat Interested	99 (29.6%)
Not Sure	85 (25.4%)
Somewhat Uninterested	26 (7.8%)
Very Uninterested	27 (8.1%)
How would you have liked to receive this exercise program(s)? (Tick all that apply)	
Face-to-face group sessions (weekly)	115 (34.4%)
Self-guided online program	87 (26.0%)
Face-to-face individual session (one off)	79 (23.7%)
Online material (e.g., videos, photos, etc.)	79 (23.7%)
Phone support	75 (22.5%)
Motivational text messages	72 (21.6%)
Online group sessions (weekly)	45 (13.5%)
Written material (e.g., pamphlets)	50 (15.0%)
Face-to-face group session (one off)	37 (11.1%)
Online individual session (one off)	30 (9.0%)
Other	23 (6.9%)
Online group session (one off)	18 (5.4%)
What type of exercise programs would you have been interested in? (Tick all that apply)	
Walking	202 (60.5%)
Yoga/Pilates	127 (38.0%)
Weights (e.g., Resistance Training, Gym)	110 (32.9%)
Swimming / Aquatic exercise	107 (32.0%)
Dancing	70 (21.0%)
Interval Training (e.g., CrossFit)	40 (12.0%)
Cycling	37 (11.1%)
Jogging	30 (9.0%)
Circuits	28 (8.4%)
Other	24 (7.5%)
While on the waiting list, how interested would you have been in being supported to complete a Healthy Eating program?	
Very Interested	25 (7.5%)
Somewhat Interested	112 (33.5%)
Not Sure	51 (15.3%)
Somewhat Uninterested	36 (10.8%)
Very Uninterested	25 (7.5%)
Missing	12 (3.6%)
How would you have liked to receive this Healthy Eating program? (Tick all that apply)	
Cooking workshops	116 (34.7%)
Online material (e.g., videos, photos, etc.)	100 (29.9%)
Written material (e.g., pamphlets)	96 (28.7%)
Self-guided online program	89 (26.6%)
Face-to-face group sessions (weekly)	73 (21.9%)
Phone support	73 (21.9%)
Face-to-face individual session (one off)	67 (20.1%)
Motivational text messages	60 (18.0%)
Online group sessions (weekly)	51 (15.3%)
Online individual session (one off)	42 (12.6%)
Supermarket tours	30 (9.0%)
Face-to-face group session (one off)	29 (8.7%)
Online group session (one off)	25 (7.5%)
Other	12 (3.6%)
While on the waiting list, how interested would you have been to be supported to do a Sleep Education program?	
Very Interested	101 (30.2%)
Somewhat Interested	89 (26.6%)
Not Sure	64 (19.2%)
Somewhat Uninterested	33 (9.9%)
Very Uninterested	31 (9.3%)
Missing	16 (4.8%)
How would you have liked to receive this Sleep Education program? (Tick all that apply)	
Online material (e.g., videos, photos, etc.)	98 (29.3%)
Self-guided online program	96 (28.7%)
Written material (e.g., pamphlets)	83 (24.9%)
Phone support	78 (23.4%)
Face-to-face 1:1 session (one off)	73 (21.9%)
Motivational text messages	60 (18.0%)
Online 1:1 session (one off)	52 (15.6%)
Face-to-face group sessions (weekly)	52 (15.6%)
Online group sessions (weekly)	39 (11.7%)
Online group session (one off)	24 (7.2%)
Face-to-face group session (one off)	25 (7.5%)
Other	13 (3.9%)
While on the waiting list, how interested would you have been in receiving additional mental health support?	
Very Interested	167 (50.0%)
Somewhat Interested	95 (28.4%)
Not Sure	36 (10.8%)
Somewhat Uninterested	8 (2.4%)
Very Uninterested	6 (1.8%)
Missing	22 (6.6%)
How would you have liked to receive this Mental Health Support program? (Tick all that apply)	
Phone support	156 (46.7%)
Online material (e.g., videos, photos, etc.)	88 (26.3%)
Self-guided online program	84 (25.1%)
Face-to-face group sessions (weekly)	83 (24.9%)
Motivational text messages	82 (24.6%)
Face-to-face 1:1 session (one off)	77 (23.1%)
Online 1:1 session (one off)	76 (22.8%)
Online group sessions (weekly)	45 (13.5%)
Written material (e.g., pamphlets)	67 (20.1%)
Face-to-face group session (one off)	29 (8.7%)
Other	18 (5.4%)
Online group session (one off)	17 (5.1%)

### Exercise program

Over half of participants were interested in an exercise program (*n* = 192, 57.4%). The most commonly expressed preference was for the program to be delivered via weekly face-to-face group sessions (*n* = 115, 34.4%) or as a self-guided online program (*n* = 87, 26.0%). The two most common modes of exercise that participants expressed interest in were walking (*n* = 202, 60.5%) and Yoga/Pilates (*n* = 127, 38.0%).

### Healthy eating program

Less than half of participants (*n* = 137, 41%) were interested in a healthy eating program. Of the participants, approximately one third expressed interest in either cooking workshops (*n* = 116, 34.7%) or online material (*n* = 100, 29.9%).

### Sleep education program

Over half participants were interested in a sleep education program (*n* = 190, 56.6%), with the most common preference being online materials (*n* = 98, 29.3%) and a self-guided online program (*n* = 96, 28.7%).

### Additional mental health support program

Most participants were interested in receiving additional mental health support (*n* = 262, 78.4%), most commonly in the form of phone support (*n* = 156, 46.7%) and online materials (*n* = 88, 26.3%).

## Subgroup analyses

Subgroup analyses were undertaken to identify differences in preferences for lifestyle programs based on gender, geographical location, Aboriginal status and LGBTQI + status. Results which were statistically significant following Holm-Bonferroni adjustment are described below and results of all the subgroup analyses are shown in Additional file 1.

### Gender

#### Exercise program

Females were more likely to be interested in yoga/Pilates and dancing compared with males (yoga/Pilates: 46.3% vs 15.9%, χ^2^ = 19.280, *φ* = 0.25, *p* < 0.01; dancing: 25.1% vs 7.9%, χ^2^ = 8.69, *φ* = 0.17*, p* < 0.01). Females were also more likely to be interested in a self-guided online exercise program (females 31.1% vs males 14.3%; χ^2^ = 7.01, *φ* = 0.13, *p* < 0.01).

#### Healthy eating program

Females were more likely to prefer online material (37.0% vs. 15.9%); χ^2^ = 10.12, *φ* = 0.18, *p* < 0.01) compared with males.

#### Additional mental health support program

Females (31.1%) were more likely than males (11.1%) to prefer an online self-guided program (χ^2^ = 10.07, *φ* = 0.18, *p* < 0.01).

### Geographical location

There were no significant differences in program preferences based on geographical location (metropolitan versus regional) following Holm-Bonferroni correction.

## Aboriginal Peoples

### Healthy eating program

Aboriginal peoples were less likely to express interest in receiving support with healthy eating (28.5% vs 66.7% for non-Aboriginal participants, χ^2^ = 33.70, *φ* = 0.34, *p* < 0.01).

#### *LGBTQI* + 

##### Exercise program

LGBTQI + participants expressed relatively more interest in swimming (56.5% vs. 30.1%; χ^2^ = 12.12, *φ* = 0.20, *p* < 0.01), dancing (43.5% vs. 17.8%; χ^2^ = 15.24, *φ* = 0.23, *p* < 0.01) and face-to-face individual sessions (43.5% vs. 20.5%; χ^2^ = 11.37, *φ* = 0.20, *p* < 0.01), compared with non-LGBTQI + participants.

##### Healthy eating program

LGBTQI + participants were more likely to express interest in cooking workshops (54.3% vs. 33.6%; χ^2^ = 7.24, *φ* = 0.16, *p* < 0.01), and one-off online individual sessions (32.6% vs. 9.7%; χ^2^ = 18.07, *φ* = 0.25, *p* < 0.01), compared with non-LGBTQI + participants.

##### Sleep education program

 LGBTQI + participants were more likely to express interest in a self-guided online program (52.2% vs. 27.4%; χ^2^ = 11.17, *φ* = 0.19, *p* < 0.01), a one-off face-to-face 1:1 session (39.1% vs. 21.2%; χ^2^ = 6.87, *φ* = 0.15, *p* < 0.01), and one-off online 1:1 sessions (34.8% vs. 13.5%; χ^2^ = 12.69, *φ* = 0.21, *p* < 0.01), compared with non-LGBTQI + participants.

## Discussion

This study set out to understand the interest in and preferred format and modes for lifestyle support for people waiting for mental health services. Nearly half of the participants (47%) reported that their mental health deteriorated whilst on the waiting list, underscoring the importance of supportive lifestyle services during the waiting period. In general, the needs assessment identified strong demand for lifestyle support, with around three in five participants requesting support for exercise and sleep, and two in five requesting support with healthy eating. In addition, the study identified a very strong demand for psychological support whilst awaiting intensive, ongoing services. Key differences in mental health symptoms and healthy lifestyle support requirements were identified based on participants’ gender, Aboriginal status, geographical location, and LGBTQI + status. Of note, persons who identify as LGBTQI + reported a greater number and more severe mental health symptoms whilst on the waiting list, and a particularly stronger desire for healthy lifestyle services. However, it’s important to consider that we didn't compare lifestyle support to other types of support. Given the greater prevalence of mental health concerns among persons who identify as LGBTQI + , it is possible that their higher levels of endorsement reflect their interest in support in general, and not specifically lifestyle support.

A clear finding from this study was that participants reported strong demand for services to support them to adopt healthy lifestyles. Our findings are consistent with previous findings involving clients of community health services in Australia, that found that individuals with a mental illness are interested in improving their health risk behaviours [17] and that providing additional preventive care consultation is acceptable to community mental health clients [18]. Australian clinical guidelines recommend improving lifestyle behaviours as a first-line treatment approach for mental health concerns [19, 20], however, in practice, pharmacological and psychotherapy-based approaches are more commonly prescribed [21] Whilst there are many self-management lifestyle programs available (both targeted at the general population, and specifically toward people with complex mental health needs) [22–24], uptake and engagement with such programs is challenging [25, 26]. This is likely to be an even bigger problem for persons living with mental health concerns, who frequently experience high rates of physical inactivity, poor nutrition, overweight and obesity [27], in addition to mental illness-related factors (e.g., amotivation, paranoid symptoms), side effects of medication (e.g., fatigue) and cognitive and psychosocial factors (e.g., social isolation or poverty) [26, 28]. Our study identified stronger demand for in-person support, particularly for exercise and diet interventions. There is a wealth of evidence supporting the effectiveness of exercise, healthy eating habits and good sleep hygiene in improving mental health symptoms [29–32], however such services are expensive, and are currently not offered routinely.

Our findings highlighted that preference for online and self-management approaches varied for the different subgroups we examined. In particular, females and participants located in regional areas were more likely to express interest in digital and self-management approaches than their male and metropolitan counterparts, respectively. A relatively higher receptiveness to these approaches amongst females is consistent with previous literature [33]. In contrast, the relatively higher interest in self-management and digital approaches observed for participants living in rural and regional areas is inconsistent with previous evidence [34]. Historically, rural, and regional areas have been slower to adopt digital approaches, and this has largely been redressed in recent years, particularly in high-income countries like Australia, which have high levels of internet and mobile coverage (except in the extremely remote areas). It is possible that regionally based individuals are accustomed to digital service access as a result of COVID-19 and the way in which persons adapted to social isolation requirements. It is also likely that the geographic gradient in willingness to adopt self-management and digital approaches may be underpinned by socioeconomic disadvantage, given that the metropolitan areas served by the organisation in this study are predominantly socioeconomically disadvantaged areas, whilst the regional areas are more socioeconomically diverse [35, 36].

Results highlighted a higher level of complexity and severity of mental health symptoms amongst persons who identify as LGBTQI + ; a cohort that is increasingly being recognised as a high need population for mental health services [37, 38]. The large number of differences observed between LGBTQI + and non-LGBTQI + populations in our study may be attributed to the higher levels of distress observed among LGBTQI + populations. Previous findings have shown that that over half (57.2%) of Australian adults who identify as LGBTQI + have high or very high levels of psychological distress, compared to 15% in the general population [5]. Encouragingly, this group expressed particularly high levels of interest in lifestyle management approaches, with a preference for face-to-face and online individual (one-on-one) sessions. These findings suggest that further work is warranted to understand the programming and delivery approaches to meet the needs of this group.

Persons who identified as Aboriginal and/or Torres Strait Islander were less likely to express interest in support with healthy eating, compared to their non-Aboriginal counterparts however there were no other significant differences between Aboriginal and non-Aboriginal participants (effect size range: *φ* = 0.00 to 0.15). Various health disparities between Aboriginal and non-Aboriginal peoples exist, with 24% of Aboriginal people being diagnosed with a mental health condition, and 31% reporting ‘high or very high’ levels of psychological distress [6]. Together, these findings suggest that further research is required to identify and implement effective and tailored services to support Aboriginal and Torres Strait Islander persons to engage in lifestyle interventions.

A key strength of the current study was that it was driven by clinicians. As a collaboration between industry and academics, we have applied rigorous, evidence-based approaches to drive a clinical quality improvement activity which will directly influence future service delivery. The survey attracted a large number of participants, which allowed us to consider the needs of several population subgroups. Limitations must also be acknowledged. The response rate was low, though similar to other patient experience surveys implemented by the organisation and reported in the health service literature [39, 40]. As a result, we had relatively small samples for some subgroup analyses (particularly for the Aboriginal status analysis). For analysis, we collapsed the 5-point Likert data into 3 categories, which aided interpretation but means that our analyses do not differentiate between strong versus moderate interest. The study was powered to detect moderate between group effect size differences, but not small effect size differences. Mental health symptoms and length of wait were retrospectively self-reported and may have been impacted by recall bias and other biases. Finally, it is unclear whether the responses obtained in this study are representative of the wider sample of clients. It is possible that more motivated individuals may have participated in the survey, which could lead to overestimation of the demand for lifestyle support services.

Given the seemingly high level of interest in lifestyle support services identified in this study, future work appears warranted to develop and pilot new services for people who are waiting for mental health support. Due to the challenges of encouraging uptake and engagement in lifestyle programs, the role of lived experiences should also play an important role in informing the development and implementation of programs in future research. This includes the involvement of Aboriginal and Torres Strait Islander people in the design and conduct of studies and identifying and implementing the supports that work best for them. Generally, in person and weekly services were most requested, with a particularly high level of interest in weekly walking groups. The effectiveness, and cost of delivering, such services will vary depending on the service component and delivery format. Therefore, it will be important to evaluate (including cost-effectiveness evaluation) any new programs to inform ongoing allocation of limited health funding resources.

In conclusion, mental health is a prevalent and growing concern. Mental health service resources are strained, contributing to extended waiting times, and potential deterioration of individuals’ conditions. There appears to be considerable desire for lifestyle support services; particularly for ongoing in-person exercise opportunities and self-directed sleep education. The interest and preferred delivery modes vary according to population subgroups, with persons who identify as LGBTQI + appearing to have relatively high mental health needs and receptiveness to lifestyle support services. Future work to rigorously develop and evaluate lifestyle support services for mental health patients, and the effects of demographic characteristics (e.g., age) are warranted.

## Supplementary Information

**Addtional file 1. **Survey responses - Subgroup analyses by gender.

## Data Availability

The datasets used and/or analysed during the current study are available from the corresponding author on reasonable request.
