# Supplementary material for: A needs assessment for self-management services for adults awaiting community-based mental health services
Source: BMC Public Health. 2023 Mar 27;23:570. doi: 10.1186/s12889-023-15382-8 (PMC10041506; doi:10.1186/s12889-023-15382-8)
Supplement: Supplementary file 1 — Addtional file 1. Survey responses - Subgroup analyses by gender. [file 12889_2023_15382_MOESM1_ESM.docx]

Additional file 1: Survey responses - Subgroup analyses by gender

|  | Gender | |  |  |  |  |
| --- | --- | --- | --- | --- | --- | --- |
|  | Female  n (%) | Male  n (%) | χ^2^ | Effect size | P value | Adjusted p value |
| **While on the waiting list, how interested would you have been to be supported to do an exercise program?** |  |  | 3.90 | 0.11 | 0.42 | 1.00 |
| Very Interested | 65 (27.8%) | 24 (38.1%) |  |  |  |  |
| Somewhat Interested | 70 (29.9%) | 17 (27.0%) |  |  |  |  |
| Not Sure | 62 (26.5%) | 12 (19.0%) |  |  |  |  |
| Somewhat Uninterested | 70 (29.9%) | 17 (27.0%) |  |  |  |  |
| Very Uninterested | 21 (9.0% | 4 (6.3%) |  |  |  |  |
| **How would you have liked to receive this exercise program(s)? (Tick all that apply)**  Face-to-face group session (one off)  Yes  No | 23 (9.8%)  212 (90.2%) | 12 (19.0%)  51 (81.0%) | 4.11 | 0.12 | 0.04 | 0.44 |
| Face-to-face group sessions (weekly)  Yes  No | 90 (38.3%)  145 (61.7%) | 17 (27.0%)  46 (73.0%) | 2.76 | 0.10 | 0.10 | 0.90 |
| Online group session (one off)  Yes  No | 11 (4.7%)  224 (95.3%) | 5 (7.9%)  58 (92.1%) | 1.04 | 0.06 | 0.31 | 1.00 |
| Online group sessions (weekly)  Yes  No | 33 (14.0%)  202 (86.0%) | 8 (12.7%)  55 (87.3%) | 0.08 | 0.02 | 0.78 | 1.00 |
| Face-to-face individual session (one off)  Yes  No | 54 (23.0%)  181 (77.0%) | 17 (27.0%)  46 (73.0%) | 0.44 | 0.04 | 0.51 | 1.00 |
| Online individual session (one off)  Yes  No | 211 (89.8%)  24 (10.2%) | 6 (9.5%)  57 (90.5%) | 0.03 | 0.01 | 0.87 | 0.87 |
| Phone support  Yes  No | 56 (23.8%)  179 (76.2%) | 17 (27.0%)  46 (73.0%) | 0.27 | 0.03 | 0.61 | 1.00 |
| Written material (e.g., pamphlets)  Yes  No | 40 (17.0%)  195 (83.0%) | 7 (11.1%)  56 (88.9%) | 1.31 | 0.07 | 0.25 | 1.00 |
| Online material (e.g., videos, photos, etc.)  Yes  No | 66 (28.1%)  169 (71.9%) | 11 (17.5%)  52 (82.5%) | 2.93 | 0.10 | 0.09 | 0.90 |
| Motivational text messages  Yes  No | 58 (24.7%)  177 (75.3%) | 11 (17.5%)  52 (82.5%) | 1.46 | 0.07 | 0.23 | 1.00 |
| Self-guided online program  Yes  No | 73 (31.1%)  162 (68.9%) | 9 (14.3%)  54 (85.7%) | 7.01 | 0.15 | **<0.01*** | **<0.01*** |
| **What type of exercise programs would you have been interested in? (Tick all that apply)**  Walking  Yes  No | 148 (63.0%)  87 (37.0%) | 40 (63.5%)  23 (36.5%) | 0.01 | 0.01 | 0.94 | 0.94 |
| Jogging  Yes  No | 21 (8.9%)  214 (91.1%) | 8 (12.7%)  55 (87.3%) | 0.80 | 0.05 | 0.37 | 1.00 |
| Interval Training (e.g., CrossFit)  Yes  No | 34 (14.5%)  201 (85.5%) | 4 (6.3%)  59 (93.7%) | 2.94 | 0.10 | 0.09 | 0.54 |
| Cycling  Yes  No | 21 (8.9%)  214 (91.1%) | 11 (17.5%)  52 (82.5%) | 3.77 | 0.11 | 0.05 | 0.35 |
| Weights (e.g., Resistance Training, Gym)  Yes  No | 77 (32.8%)  158 (67.2%) | 25 (39.7%)  38 (60.3%) | 1.06 | 0.06 | 0.30 | 1.00 |
| Swimming / Aquatic exercise  Yes  No | 80 (34.0%)  155 (66.0%) | 19 (30.2%)  44 (69.8%) | 0.34 | 0.03 | 0.56 | 1.00 |
| Yoga/Pilates  Yes  No | 109 (46.4%)  126 (53.6%) | 10 (15.9%)  53 (84.1%) | 19.28 | 0.25 | **<0.01*** | **<0.01*** |
| Circuits  Yes  No | 21 (8.9%)  214 (91.1%) | 6 (9.5%)  57 (90.5%) | 0.02 | 0.01 | 0.89 | 1.00 |
| Dancing  Yes  No | 59 (25.1%)  176 (74.9%) | 5 (7.9%)  58 (92.1%) | 8.69 | 0.17 | **<0.01*** | **<0.01*** |
| **While on the waiting list, how interested would you have been in being supported to complete a Healthy Eating program?** |  |  | 1.03 | 0.06 | 0.96 | 0.96 |
| Very Interested | 85 (36.2%) | 21 (33.3%) |  |  |  |  |
| Somewhat Interested | 70 (29.8%) | 19 (30.2%) |  |  |  |  |
| Not Sure | 33 (14.0%) | 11 (17.5%) |  |  |  |  |
| Somewhat Uninterested | 27 (11.5%) | 8 (12.7%) |  |  |  |  |
| Very Uninterested | 19 (8.1%) | 4 (6.3%) |  |  |  |  |
| Missing | 1 (0.4%) | 0 (0.0%) |  |  |  |  |
| **How would you have liked to receive this Healthy Eating program? (Tick all that apply)**  Face-to-face group session (one off)  Yes  No | 21 (8.9%)  214 (91.1%) | 7 (11.1%)  56 (88.9%) | 0.28 | 0.03 | 0.60 | 1.00 |
| Face-to-face group sessions (weekly)  Yes  No | 52 (22.1%)  183 (77.9%) | 16 (25.4%)  47 (74.6%) | 0.30 | 0.03 | 0.58 | 1.00 |
| Online group session (one off)  Yes  No | 21 (8.9%)  214 (91.1%) | 4 (6.3%)  59 (93.7%) | 0.43 | 0.04 | 0.51 | 1.00 |
| Online group sessions (weekly)  Yes  No | 42 (17.9%)  193 (82.1%) | 6 (9.5%)  57 (90.5%) | 2.56 | 0.09 | 0.11 | 1.00 |
| Face-to-face individual session (one off)  Yes  No | 52 (22.1%)  183 (77.9%) | 10 (15.9%)  53 (84.1%) | 1.18 | 0.06 | 0.28 | 1.00 |
| Online individual session (one off)  Yes  No | 35 (14.9%)  200 (85.1%) | 4 (6.3%)  59 (93.7%) | 3.19 | 0.10 | 0.07 | 0.84 |
| Phone support  Yes  No | 55 (23.4%)  180 (76.6%) | 15 (23.8%)  48 (76.2%) | 0.01 | 0.01 | 0.95 | 1.00 |
| Written material (e.g., pamphlets)  Yes  No | 76 (32.3%)  159 (67.7%) | 15 (23.8%)  48 (76.2%) | 1.71 | 0.08 | 0.19 | 1.00 |
| Online material (e.g., videos, photos, etc.)  Yes  No | 87 (37.0%)  148 (63.0%) | 10 (15.9%)  53 (84.1%) | 10.12 | 0.18 | **<0.01*** | **<0.01*** |
| Motivational text messages  Yes  No | 47 (20.0%)  188 (80.0%) | 9 (14.3%)  54 (85.7%) | 1.06 | 0.06 | 0.30 | 1.00 |
| Cooking workshops  Yes  No | 89 (37.9%)  146 (62.1%) | 20 (31.7%)  43 (68.3%) | 0.80 | 0.05 | 0.37 | 1.00 |
| Supermarket tours  Yes  No | 23 (9.8%)  212 (90.2%) | 5 (7.9%)  58 (92.1%) | 0.20 | 0.03 | 0.66 | 1.00 |
| Self-guided online program  Yes  No | 71 (30.2%)  164 (69.8%) | 10 (15.9%)  53 (84.1%) | 5.16 | 0.13 | 0.02 | 0.26 |
| **While on the waiting list, how interested would you have been to be supported to do a Sleep Education program?** |  |  | 6.97 | 0.15 | 0.22 | 1.00 |
| Very Interested | 76 (32.3%) | 19 (30.2%) |  |  |  |  |
| Somewhat Interested | 61 (26.0%) | 22 (34.9%) |  |  |  |  |
| Not Sure | 49 (20.9%) | 11 (17.5%) |  |  |  |  |
| Somewhat Uninterested | 28 (11.9%) | 4 (6.3%) |  |  |  |  |
| Very Uninterested | 21 (8.9%) | 6 (9.5%) |  |  |  |  |
| Missing | 0 (0.0%) | 1 (1.6%) |  |  |  |  |
| **How would you have liked to receive this Sleep Education program? (Tick all that apply)**  Face-to-face group session (one off)  Yes  No | 19 (8.1%)  216 (91.9%) | 6 (9.5%)  57 (90.5%) | 0.13 | 0.02 | 0.72 | 1.00 |
| Face-to-face group sessions (weekly)  Yes  No | 38 (16.2%)  197 (83.8%) | 12 (19.0%)  51 (81.0%) | 0.30 | 0.03 | 0.59 | 1.00 |
| Online group session (one off)  Yes  No | 22 (9.4%)  213 (90.6%) | 1 (1.6%)  62 (98.4%) | 4.22 | 0.12 | 0.04 | 0.44 |
| Online group sessions (weekly)  Yes  No | 34 (14.5%)  201 (85.5%) | 5 (7.9%)  58 (92.1%) | 1.86 | 0.08 | 0.17 | 1.00 |
| Face-to-face 1:1 session (one off)  Yes  No | 57 (24.3%)  178 (75.7%) | 11 (17.5%)  52 (82.5%) | 1.30 | 0.07 | 0.25 | 1.00 |
| Online 1:1 session (one off)  Yes  No | 40 (17.0%  195 (83.0% | 10 (15.9%)  53 (84.1%) | 0.05 | 0.01 | 0.83 | 1.00 |
| Phone support  Yes  No | 55 (23.4%)  180 (76.6%) | 19 (30.2%)  44 (69.8%) | 1.21 | 0.06 | 0.27 | 1.00 |
| Written material (e.g., pamphlets)  Yes  No | 62 (26.4%)  173 (73.6%) | 17 (27.0%)  46 (73.0%) | 0.01 | 0.01 | 0.92 | 0.92 |
| Online material (e.g., videos, photos, etc.)  Yes  No | 83 (35.3%)  152 (64.7%) | 12 (19.0%)  51 (81.0%) | 6.06 | 0.14 | 0.01 | 0.12 |
| Motivational text messages  Yes  No | 44 (18.7%)  191 (81.3%) | 14 (22.2%)  49 (77.8%) | 0.39 | 0.04 | 0.53 | 1.00 |
| Self-guided online program  Yes  No | 79 (33.6%)  156 (66.4%) | 13 (20.6%)  50 (79.4%) | 3.92 | 0.11 | 0.05 | 0.50 |
| **While on the waiting list, how interested would you have been to be supported to do receive additional mental health support?** |  |  | 5.14 | 0.13 | 0.27 | 1.00 |
| Very Interested | 121 (51.5%) | 38 (60.3%) |  |  |  |  |
| Somewhat Interested | 74 (31.5%) | 16 (25.4%) |  |  |  |  |
| Not Sure | 27 (11.5%) | 9 (14.3%) |  |  |  |  |
| Somewhat Uninterested | 8 (3.4%) | 0 (0.0%) |  |  |  |  |
| Very Uninterested | 5 (2.1%) | 0 (0.0%) |  |  |  |  |
| **How would you have liked to receive this Mental Health support program? (Tick all that apply)**  Face-to-face group session (one off)  Yes  No | 22 (9.4%)  213 (90.6%) | 7 (11.1%)  56 (88.9%) | 0.17 | 0.02 | 0.68 | 1.00 |
| Face-to-face group sessions (weekly)  Yes  No | 60 (25.5%)  175 (74.5%) | 19 (30.2%)  44 (69.8%) | 0.55 | 0.04 | 0.46 | 1.00 |
| Online group session (one off)  Yes  No | 12 (5.1%)  223 (94.9%) | 5 (7.9%)  58 (92.1%) | 0.74 | 0.05 | 0.39 | 1.00 |
| Online group sessions (weekly)  Yes  No | 31 (13.2%)  204 (86.8%) | 11 (17.5%)  52 (82.5%) | 0.75 | 0.05 | 0.39 | 1.00 |
| Face-to-face 1:1 session (one off)  Yes  No | 54 (23.0%)  181 (77.0%) | 19 (30.2%)  44 (69.8%) | 1.39 | 0.07 | 0.24 | 1.00 |
| Online 1:1 session (one off)  Yes  No | 61 (26.0%)  174 (74.0%) | 13 (20.6%)  50 (79.4%) | 0.75 | 0.05 | 0.39 | 1.00 |
| Phone support  Yes  No | 123 (52.3%)  112 (47.7%) | 28 (44.4%)  35 (55.6%) | 1.24 | 0.06 | 0.27 | 1.00 |
| Written material (e.g., pamphlets)  Yes  No | 51 (21.7%)  184 (78.3%) | 14 (22.2%)  49 (77.8%) | 0.01 | 0.01 | 0.93 | 0.93 |
| Online material (e.g., videos, photos, etc.)  Yes  No | 73 (31.1%)  162 (68.9%) | 12 (19.0%)  51 (81.0%) | 3.52 | 0.11 | 0.06 | 0.66 |
| Motivational text messages  Yes  No | 63 (26.8%)  172 (73.2%) | 16 (25.4%)  47 (74.6%) | 0.05 | 0.01 | 0.82 | 1.00 |
| Self-guided online program  Yes  No | 73 (31.1%)  162 (68.9%) | 7 (11.1%)  56 (88.9%) | 10.07 | 0.18 | **<0.01*** | **<0.01*** |
| Note: Tables represents results of all subgroup analyses for gender, comparing differences between males and females in responses, using Chi square tests. For all subgroup analyses, the Holm-Bonferroni correction was applied to adjust for multiple comparisons. | | | | | | |

Additional file 2: Survey responses - Subgroup analyses by location

|  | Location | |  |  |  |  |
| --- | --- | --- | --- | --- | --- | --- |
|  | Country | Metro | χ^2^ | Effect size | P value | Adjusted p value |
| **While on the waiting list, how interested would you have been to be supported to do an exercise program?** |  |  | 1.55 | 0.07 | 0.91 | 1.00 |
| Very Interested | 14 (24.6%) | 79 (28.8%) |  |  |  |  |
| Somewhat Interested | 19 (33.3%) | 80 (29.2%) |  |  |  |  |
| Not Sure | 14 (24.6%) | 71 (25.9%) |  |  |  |  |
| Somewhat Uninterested | 6 (10.5%) | 20 (7.3%) |  |  |  |  |
| Very Uninterested | 4 (7.0%) | 23 (8.4%) |  |  |  |  |
| Missing | 0 (0.0%) | 1 (0.4%) |  |  |  |  |
| **How would you have liked to receive this exercise program(s)? (Tick all that apply)**  Face-to-face group session (one off)  Yes  No | 7 (12.3%)  50 (87.7%) | 30 (10.8%)  247 (89.2%) | 0.10 | 0.02 | 0.75 | 1.00 |
| Face-to-face group sessions (weekly)  Yes  No | 22 (38.6%)  35 (61.4%) | 93 (33.6%)  184 (66.4%) | 0.53 | 0.04 | 0.47 | 1.00 |
| Online group session (one off)  Yes  No | 3 (5.3%)  54 (94.7%) | 15 (5.4%)  262 (94.6%) | 0.00 | 0.00 | 0.96 | 0.96 |
| Online group sessions (weekly)  Yes  No | 3 (5.3%)  54 (94.7%) | 42 (15.2%)  235 (84.8%) | 3.97 | 0.12 | 0.05 | 0.60 |
| Face-to-face individual session (one off)  Yes  No | 15 (26.3%)  42 (73.7%) | 64 (23.1%)  213 (76.9%) | 0.27 | 0.03 | 0.60 | 1.00 |
| Online individual session (one off)  Yes  No | 2 (3.5%)  55 (96.5%) | 28 (10.1%)  249 (89.9%) | 2.52 | 0.09 | 0.11 | 1.00 |
| Phone support  Yes  No | 13 (22.8%)  44 (77.2%) | 62 (22.4%)  215 (77.6%) | 0.01 | 0.01 | 0.94 | 1.00 |
| Written material (e.g., pamphlets)  Yes  No | 7 (12.3%)  50 (87.7%) | 43 (15.5%)  234 (84.5%) | 0.39 | 0.04 | 0.53 | 1.00 |
| Online material (e.g., videos, photos, etc.)  Yes  No | 12 (21.1%)  45 (78.9%) | 67 (24.2%)  210 (75.8%) | 0.26 | 0.03 | 0.61 | 1.00 |
| Motivational text messages  Yes  No | 16 (28.1%)  41 (71.9%) | 56 (20.2%)  221 (79.8%) | 1.72 | 0.08 | 0.19 | 1.00 |
| Self-guided online program  Yes  No | 16 (28.1%)  41 (71.9%) | 71 (25.6%)  206 (74.4%) | 0.15 | 0.02 | 0.70 | 1.00 |
| **What type of exercise programs would you have been interested in? (Tick all that apply)**  Walking  Yes  No | 30 (52.6%)  27 (47.4%) | 172 (62.1%)  105 (37.9%) | 1.77 | 0.08 | 0.18 | 1.00 |
| Jogging  Yes  No | 4 (7.0%)  53 (93.0%) | 26 (9.4%)  251 (90.6%) | 0.32 | 0.03 | 0.57 | 1.00 |
| Interval Training (e.g., CrossFit)  Yes  No | 8 (14.0%)  49 (86.0%) | 32 (11.6%)  245 (88.4%) | 0.28 | 0.03 | 0.60 | 1.00 |
| Cycling  Yes  No | 4 (7.0%)  53 (93.0%) | 33 (11.9%)  244 (88.1%) | 1.15 | 0.06 | 0.28 | 1.00 |
| Weights (e.g., Resistance Training, Gym)  Yes  No | 18 (31.6%)  39 (68.4%) | 92 (33.2%)  185 (66.8%) | 0.06 | 0.01 | 0.81 | 0.81 |
| Swimming / Aquatic exercise  Yes  No | 14 (24.6%)  43 (75.4%) | 93 (33.6%)  184 (66.4%) | 1.76 | 0.08 | 0.18 | 1.00 |
| Yoga/Pilates  Yes  No | 26 (45.6%)  31 (54.4%) | 101 (36.5%)  176 (63.5%) | 1.68 | 0.08 | 0.20 | 1.00 |
| Circuits  Yes  No | 3 (5.3%)  54 (94.7%) | 25 (9.0%)  252 (91.0%) | 0.87 | 0.05 | 0.35 | 1.00 |
| Dancing  Yes  No | 15 (26.3%)  42 (73.7%) | 55 (19.9%)  222 (80.1%) | 1.19 | 0.06 | 0.28 | 1.00 |
| **While on the waiting list, how interested would you have been in being supported to complete a Healthy Eating program?** |  |  | 0.25 | 0.03 | 1.00 | 1.00 |
| Very Interested | 18 (31.6%) | 94 (33.9%) |  |  |  |  |
| Somewhat Interested | 17 (29.8%) | 81 (29.2%) |  |  |  |  |
| Not Sure | 9 (15.8%) | 42 (15.2%) |  |  |  |  |
| Somewhat Uninterested | 6 (10.5%) | 30 (10.8%) |  |  |  |  |
| Very Uninterested | 5 (8.8%) | 20 (7.2%) |  |  |  |  |
| Missing | 2 (3.5%) | 10 (3.6%) |  |  |  |  |
| **How would you have liked to receive this Healthy Eating program? (Tick all that apply)**  Face-to-face group session (one off)  Yes  No | 4 (7.0%)  53 (93.0%) | 25 (9.0%)  252 (91.0%) | 0.24 | 0.03 | 0.62 | 1.00 |
| Face-to-face group sessions (weekly)  Yes  No | 9 (15.8%)  48 (84.2%) | 64 (23.1%)  213 (76.9%) | 1.48 | 0.07 | 0.22 | 1.00 |
| Online group session (one off)  Yes  No | 1 (1.8%)  56 (98.2%) | 24 (8.7%)  253 (91.3%) | 3.26 | 0.10 | 0.07 | 0.91 |
| Online group sessions (weekly)  Yes  No | 6 (10.5%)  51 (89.5%) | 45 (16.2%)  232 (83.8%) | 1.20 | 0.06 | 0.27 | 1.00 |
| Face-to-face individual session (one off)  Yes  No | 11 (19.3%)  46 (80.7%) | 56 (20.2%)  221 (79.8%) | 0.03 | 0.01 | 0.88 | 1.00 |
| Online individual session (one off)  Yes  No | 3 (5.3%)  54 (94.7%) | 39 (14.1%)  238 (85.9%) | 3.34 | 0.11 | 0.07 | 0.91 |
| Phone support  Yes  No | 13 (22.8%)  44 (77.2%) | 60 (21.7%)  217 (78.3%) | 0.04 | 0.01 | 0.85 | 1.00 |
| Written material (e.g., pamphlets)  Yes  No | 17 (29.8%)  40 (70.2%) | 79 (28.5%)  198 (71.5%) | 0.04 | 0.01 | 0.84 | 1.00 |
| Online material (e.g., videos, photos, etc.)  Yes  No | 18 (31.6%)  39 (68.4%) | 82 (29.6%)  195 (70.4%) | 0.09 | 0.02 | 0.77 | 1.00 |
| Motivational text messages  Yes  No | 7 (12.3%)  50 (87.7%) | 53 (19.1%)  224 (80.9%) | 1.51 | 0.07 | 0.22 | 1.00 |
| Cooking workshops  Yes  No | 20 (35.1%)  37 (64.9%) | 96 (34.7%)  181 (65.3%) | 0.00 | 0.00 | 0.95 | 1.00 |
| Supermarket tours  Yes  No | 4 (7.0%)  53 (93.0%) | 26 (9.4%)  251 (90.6%) | 0.32 | 0.03 | 0.57 | 1.00 |
| Self-guided online program  Yes  No | 17 (29.8%)  40 (70.2%) | 72 (26.0%)  205 (74.0%) | 0.36 | 0.03 | 0.55 | 1.00 |
| **While on the waiting list, how interested would you have been to be supported to do a Sleep Education program?** |  |  | 4.33 | 0.12 | 0.50 | 1.00 |
| Very Interested | 19 (33.3%) | 82 (29.6%) |  |  |  |  |
| Somewhat Interested | 19 (33.3%) | 70 (25.3%) |  |  |  |  |
| Not Sure | 7 (12.3%) | 57 (20.6%) |  |  |  |  |
| Somewhat Uninterested | 6 (10.5%) | 27 (9.7%) |  |  |  |  |
| Very Uninterested | 3 (5.3%) | 28 (10.1%) |  |  |  |  |
| Missing | 3 (5.3%) | 13 (4.7%) |  |  |  |  |
| **How would you have liked to receive this Sleep Education program? (Tick all that apply)**  Face-to-face group session (one off)  Yes  No | 6 (10.5%)  51 (89.5%) | 19 (6.9%)  258 (93.1%) | 0.92 | 0.06 | 0.34 | 1.00 |
| Face-to-face group sessions (weekly)  Yes  No | 6 (10.5%)  51 (89.5%) | 46 (16.6%)  231 (83.4%) | 1.33 | 0.07 | 0.25 | 1.00 |
| Online group session (one off)  Yes  No | 3 (5.3%)  54 (94.7%) | 21 (7.6%)  256 (92.4%) | 0.38 | 0.04 | 0.54 | 1.00 |
| Online group sessions (weekly)  Yes  No | 3 (5.3%)  54 (94.7%) | 36 (13.0%)  241 (87.0%) | 2.74 | 0.10 | 0.10 | 1.00 |
| Face-to-face 1:1 session (one off)  Yes  No | 14 (24.6%)  43 (75.4%) | 59 (21.3%)  218 (78.7%) | 0.29 | 0.03 | 0.59 | 1.00 |
| Online 1:1 session (one off)  Yes  No | 7 (12.3%)  50 (87.7%) | 45 (16.2%)  232 (83.8%) | 0.57 | 0.04 | 0.45 | 1.00 |
| Phone support  Yes  No | 13 (22.8%)  44 (77.2%) | 65 (23.5%)  212 (76.5%) | 0.01 | 0.01 | 0.92 | 0.92 |
| Written material (e.g., pamphlets)  Yes  No | 12 (21.1%)  45 (78.9%) | 71 (25.6%)  206 (74.4%) | 0.53 | 0.04 | 0.47 | 1.00 |
| Online material (e.g., videos, photos, etc.)  Yes  No | 23 (40.4%)  34 (59.6%) | 75 (27.1%)  202 (72.9%) | 4.02 | 0.12 | 0.05 | 0.60 |
| Motivational text messages  Yes  No | 11 (19.3%)  46 (80.7%) | 49 (17.7%)  228 (82.3%) | 0.08 | 0.02 | 0.77 | 1.00 |
| Self-guided online program  Yes  No | 17 (29.8%)  40 (70.2%) | 79 (28.5%)  198 (71.5%) | 0.04 | 0.01 | 0.84 | 1.00 |
| **While on the waiting list, how interested would you have been to be supported to do receive additional mental health support?** |  |  | 3.52 | 0.11 | 0.62 | 1.00 |
| Very Interested | 27 (47.4%) | 140 (50.5%) |  |  |  |  |
| Somewhat Interested | 14 (24.6%) | 81 (29.2%) |  |  |  |  |
| Not Sure | 8 (14.0%) | 28 (10.1%) |  |  |  |  |
| Somewhat Uninterested | 3 (5.3%) | 5 (1.8%) |  |  |  |  |
| Very Uninterested | 1 (1.8%) | 5 (1.8%) |  |  |  |  |
| Missing | 4 (7.0%) | 18 (6.5%) |  |  |  |  |
| **How would you have liked to receive this Mental Health support program? (Tick all that apply)**  Face-to-face group session (one off)  Yes  No | 7 (12.3%)  50 (87.7%) | 22 (7.9%)  255 (92.1%) | 1.12 | 0.06 | 0.29 | 1.00 |
| Face-to-face group sessions (weekly)  Yes  No | 13 (22.8%)  44 (77.2%) | 70 (25.3%)  207 (74.7%) | 0.15 | 0.02 | 0.70 | 1.00 |
| Online group session (one off)  Yes  No | 3 (5.3%)  54 (94.7%) | 14 (5.1%)  263 (94.9%) | 0.00 | 0.00 | 0.95 | 1.00 |
| Online group sessions (weekly)  Yes  No | 5 (8.8%)  52 (91.2%) | 40 (14.4%)  237 (85.6%) | 1.30 | 0.07 | 0.25 | 1.00 |
| Face-to-face 1:1 session (one off)  Yes  No | 16 (28.1%)  41 (71.9%) | 61 (22.0%)  216 (78.0%) | 0.98 | 0.06 | 0.32 | 1.00 |
| Online 1:1 session (one off)  Yes  No | 11 (19.3%)  46 (80.7%) | 65 (23.5%)  212 (76.5%) | 0.47 | 0.04 | 0.49 | 1.00 |
| Phone support  Yes  No | 26 (45.6%)  31 (54.4%) | 130 (46.9%)  147 (53.1%) | 0.03 | 0.01 | 0.86 | 1.00 |
| Written material (e.g., pamphlets)  Yes  No | 12 (21.1%)  45 (78.9%) | 55 (19.9%)  222 (80.1%) | 0.04 | 0.01 | 0.84 | 1.00 |
| Online material (e.g., videos, photos, etc.)  Yes  No | 19 (33.3%)  38 (66.7%) | 69 (24.9%)  208 (75.1%) | 1.73 | 0.08 | 0.19 | 1.00 |
| Motivational text messages  Yes  No | 14 (24.6%)  43 (75.4%) | 68 (24.5%)  209 (75.5%) | 0.00 | 0.00 | 1.00 | 1.00 |
| Self-guided online program  Yes  No | 16 (28.1%)  41 (71.9%) | 68 (24.5%)  209 (75.5%) | 0.31 | 0.03 | 0.58 | 1.00 |
| Note: This table shows results of all subgroup analyses for residence, comparing differences between males and females in responses, using Chi square tests. For all subgroup analyses, the Holm-Bonferroni correction was applied to adjust for multiple comparisons. | | | | | | |

Additional file 3: Survey responses - Subgroup analyses by ATSI status

|  | ATSI status | |  |  |  |  |
| --- | --- | --- | --- | --- | --- | --- |
|  | YES | NO | χ^2^ | Effect size | P value | Adjusted p value |
| **While on the waiting list, how interested would you have been to be supported to do an exercise program?** |  |  | 1.99 | 0.08 | 0.74 | 0.74 |
| Very Interested | 5 (35.7%) | 86 (29.6%) |  |  |  |  |
| Somewhat Interested | 2 (14.3%) | 88 (30.2%) |  |  |  |  |
| Not Sure | 4 (28.6%) | 71 (24.4%) |  |  |  |  |
| Somewhat Uninterested | 1 (7.1%) | 22 (7.6%) |  |  |  |  |
| Very Uninterested | 2 (14.3%) | 24 (8.2%) |  |  |  |  |
| **How would you have liked to receive this exercise program(s)? (Tick all that apply)**  Face-to-face group session (one off)  Yes  No | 1 (7.1%)  13 (92.9%) | 34 (11.6%)  258 (88.4%) | 0.27 | 0.03 | 0.61 | 1.00 |
| Face-to-face group sessions (weekly)  Yes  No | 3 (21.4%)  11 (78.6%) | 108 (37.0%)  184 (63.0%) | 1.40 | 0.07 | 0.24 | 1.00 |
| Online group session (one off)  Yes  No | 0 (0.0%)  14 (100.0%) | 16 (5.5%)  276 (94.5%) | 0.81 | 0.05 | 0.37 | 1.00 |
| Online group sessions (weekly)  Yes  No | 1 (7.1%)  13 (92.9%) | 42 (14.4%)  250 (85.6%) | 0.58 | 0.04 | 0.45 | 1.00 |
| Face-to-face individual session (one off)  Yes  No | 4 (28.6%)  10 (71.4%) | 70 (24.0%)  222 (76.0%) | 0.15 | 0.02 | 0.70 | 1.00 |
| Online individual session (one off)  Yes  No | 3 (21.4%)  11 (78.6%) | 27 (9.2%)  265 (90.8%) | 2.24 | 0.09 | 0.13 | 1.00 |
| Phone support  Yes  No | 6 (42.9%)  8 (57.1%) | 68 (23.3%)  224 (76.7%) | 2.79 | 0.10 | 0.10 | 1.00 |
| Written material (e.g., pamphlets)  Yes  No | 3 (21.4%)  11 (78.6%) | 45 (15.4%)  247 (84.6%) | 0.37 | 0.04 | 0.55 | 1.00 |
| Online material (e.g., videos, photos, etc.)  Yes  No | 3 (21.4%)  11 (78.6%) | 75 (25.7%)  217 (74.3%) | 0.13 | 0.02 | 0.72 | 1.00 |
| Motivational text messages  Yes  No | 2 (14.3%)  12 (85.7%) | 70 (24.0%)  222 (76.0%) | 0.70 | 0.05 | 0.40 | 1.00 |
| Self-guided online program  Yes  No | 3 (21.4%)  11 (78.6%) | 82 (28.1%)  210 (71.9%) | 0.30 | 0.03 | 0.59 | 1.00 |
| **What type of exercise programs would you have been interested in? (Tick all that apply)**  Walking  Yes  No | 9 (64.3%)  5 (35.7%) | 182 (62.3%)  110 (37.7%) | 0.02 | 0.01 | 0.88 | 1.00 |
| Jogging  Yes  No | 0 (0.0%)  14 (100.0%) | 29 (9.9%)  263 (90.1%) | 1.54 | 0.07 | 0.22 | 1.00 |
| Interval Training (e.g., CrossFit)  Yes  No | 0 (0.0%)  14 (100.0%) | 38 (13.0%)  254 (87.0%) | 2.08 | 0.08 | 0.15 | 1.00 |
| Cycling  Yes  No | 1 (7.1%)  13 (92.9%) | 35 (12.0%)  257 (88.0%) | 0.30 | 0.03 | 0.58 | 1.00 |
| Weights (e.g., Resistance Training, Gym)  Yes  No | 5 (35.7%)  9 (64.3%) | 100 (34.2%)  192 (65.8%) | 0.01 | 0.01 | 0.91 | 0.91 |
| Swimming / Aquatic exercise  Yes  No | 3 (21.4%)  11 (78.6%) | 102 (34.9%)  190 (65.1%) | 1.08 | 0.06 | 0.30 | 1.00 |
| Yoga/Pilates  Yes  No | 5 (35.7%)  9 (64.3%) | 118 (40.4%)  174 (59.6%) | 0.12 | 0.02 | 0.73 | 1.00 |
| Circuits  Yes  No | 2 (14.3%)  12 (85.7%) | 25 (8.6%)  267 (91.4%) | 0.54 | 0.04 | 0.46 | 1.00 |
| Dancing  Yes  No | 4 (28.6%)  10 (71.4%) | 63 (21.6%)  229 (78.4%) | 0.38 | 0.04 | 0.54 | 1.00 |
| **While on the waiting list, how interested would you have been in being supported to complete a Healthy Eating program?**  Very Interested | 1 (7.1%) | 107 (36.6%) | 33.70 | 0.34 | **<0.01*** | **<0.01*** |
| Somewhat Interested | 3 (21.4%) | 88 (30.1%) |  |  |  |  |
| Not Sure | 2 (14.3%) | 44 (15.1%) |  |  |  |  |
| Somewhat Uninterested | 3 (21.4%) | 33 (11.3%) |  |  |  |  |
| Very Uninterested | 4 (28.6%) | 20 (6.8%) |  |  |  |  |
| Missing | 1 (7.1%) | 0 (0.0%) |  |  |  |  |
| **How would you have liked to receive this Healthy Eating program? (Tick all that apply)**  Face-to-face group session (one off)  Yes  No | 1 (7.1%)  13 (92.9%) | 27 (9.2%)  265 (90.8%) | 0.07 | 0.02 | 0.79 | 1.00 |
| Face-to-face group sessions (weekly)  Yes  No | 1 (7.1%)  13 (92.9%) | 69 (23.6%)  223 (76.4%) | 2.06 | 0.08 | 0.15 | 1.00 |
| Online group session (one off)  Yes  No | 1 (7.1%)  13 (92.9%) | 24 (8.2%)  268 (91.8%) | 0.02 | 0.01 | 0.89 | 0.89 |
| Online group sessions (weekly)  Yes  No | 1 (7.1%)  13 (92.9%) | 48 (16.4%)  244 (83.6%) | 0.86 | 0.05 | 0.35 | 1.00 |
| Face-to-face individual session (one off)  Yes  No | 1 (7.1%)  13 (92.9%) | 64 (21.9%)  228 (78.1%) | 1.74 | 0.08 | 0.19 | 1.00 |
| Online individual session (one off)  Yes  No | 1 (7.1%)  13 (92.9%) | 39 (13.4%)  253 (86.6%) | 0.45 | 0.04 | 0.50 | 1.00 |
| Phone support  Yes  No | 2 (14.3%)  12 (85.7%) | 69 (23.6%)  223 (76.4%) | 0.66 | 0.05 | 0.42 | 1.00 |
| Written material (e.g., pamphlets)  Yes  No | 3 (21.4%)  11 (78.6%) | 89 (30.5%)  203 (69.5%) | 0.52 | 0.04 | 0.47 | 1.00 |
| Online material (e.g., videos, photos, etc.)  Yes  No | 1 (7.1%)  13 (92.9%) | 97 (33.2%)  195 (66.8%) | 4.17 | 0.12 | 0.04 | 0.44 |
| Motivational text messages  Yes  No | 0 (0.0%)  14 (100.0%) | 57 (19.5%)  235 (80.5%) | 3.36 | 0.11 | 0.07 | 0.70 |
| Cooking workshops  Yes  No | 1 (7.1%)  13 (92.9%) | 112 (38.4%)  180 (61.6%) | 5.59 | 0.14 | 0.02 | 0.24 |
| Supermarket tours  Yes  No | 0 (0.0%)  14 (100.0%) | 30 (10.3%)  262 (89.7%) | 1.60 | 0.07 | 0.21 | 1.00 |
| Self-guided online program  Yes  No | 0 (0.0%)  14 (100.0%) | 84 (28.8%)  208 (71.2%) | 5.55 | 0.14 | 0.02 | 0.24 |
| **While on the waiting list, how interested would you have been to be supported to do a Sleep Education program?**  Very Interested | 5 (35.7%) | 92 (31.5%) | 2.20 | 0.09 | 0.82 | 1.00 |
| Somewhat Interested | 5 (35.7%) | 80 (27.4%) |  |  |  |  |
| Not Sure | 1 (7.1%) | 60 (20.5%) |  |  |  |  |
| Somewhat Uninterested | 1 (7.1%) | 32 (11.0%) |  |  |  |  |
| Very Uninterested | 2 (14.3%) | 27 (9.2%) |  |  |  |  |
| Missing | 0 (0.0%) | 1 (0.3%) |  |  |  |  |
| **How would you have liked to receive this Sleep Education program? (Tick all that apply)**  Face-to-face group session (one off)  Yes  No | 0 (0.0%)  14 (100.0%) | 25 (8.6%)  267 (91.4%) | 1.31 | 0.07 | 0.25 | 1.00 |
| Face-to-face group sessions (weekly)  Yes  No | 3 (21.4%)  11 (78.6%) | 49 (16.8%)  243 (83.2%) | 0.21 | 0.03 | 0.65 | 1.00 |
| Online group session (one off)  Yes  No | 1 (7.1%)  13 (92.9%) | 22 (7.5%)  270 (92.5%) | 0.00 | 0.00 | 0.96 | 0.96 |
| Online group sessions (weekly)  Yes  No | 1 (7.1%)  13 (92.9%) | 38 (13.0%)  254 (87.0%) | 0.41 | 0.04 | 0.52 | 1.00 |
| Face-to-face 1:1 session (one off)  Yes  No | 4 (28.6%)  10 (71.4%) | 68 (23.3%)  224 (76.7%) | 0.21 | 0.03 | 0.65 | 1.00 |
| Online 1:1 session (one off)  Yes  No | 1 (7.1%)  13 (92.9%) | 50 (17.1%)  242 (82.9%) | 0.96 | 0.06 | 0.33 | 1.00 |
| Phone support  Yes  No | 4 (28.6%)  10 (71.4%) | 72 (24.7%)  220 (75.3%) | 0.11 | 0.02 | 0.74 | 1.00 |
| Written material (e.g., pamphlets)  Yes  No | 5 (35.7%)  9 (64.3%) | 77 (26.4%)  215 (73.6%) | 0.60 | 0.04 | 0.44 | 1.00 |
| Online material (e.g., videos, photos, etc.)  Yes  No | 4 (28.6%)  10 (71.4%) | 92 (31.5%)  200 (68.5%) | 0.05 | 0.01 | 0.82 | 1.00 |
| Motivational text messages  Yes  No | 1 (7.1%)  13 (92.9%) | 58 (19.9%)  234 (80.1%) | 1.39 | 0.07 | 0.24 | 1.00 |
| Self-guided online program  Yes  No | 2 (14.3%)  12 (85.7%) | 94 (32.2%)  198 (67.8%) | 1.99 | 0.08 | 0.16 | 1.00 |
| **While on the waiting list, how interested would you have been to be supported to do receive additional mental health support?**  Very Interested | 8 (57.1%) | 157 (53.8%) | 1.14 | 0.06 | 0.89 | 1.00 |
| Somewhat Interested | 5 (35.7%) | 86 (29.5%) |  |  |  |  |
| Not Sure | 1 (7.1%) | 35 (12.0%) |  |  |  |  |
| Somewhat Uninterested | 0 (0.0%) | 8 (2.7%) |  |  |  |  |
| Very Uninterested | 0 (0.0%) | 6 (2.1%) |  |  |  |  |
| **How would you have liked to receive this Mental Health support program? (Tick all that apply)**  Face-to-face group session (one off)  Yes  No | 0 (0.0%)  14 (100.0%) | 29 (9.9%)  263 (90.1%) | 1.54 | 0.07 | 0.22 | 1.00 |
| Face-to-face group sessions (weekly)  Yes  No | 2 (14.3%)  12 (85.7%) | 80 (27.4%)  212 (72.6%) | 1.17 | 0.06 | 0.28 | 1.00 |
| Online group session (one off)  Yes  No | 0 (0.0%)  14 (100.0%) | 17 (5.8%)  275 (94.2%) | 0.86 | 0.05 | 0.35 | 1.00 |
| Online group sessions (weekly)  Yes  No | 1 (7.1%)  13 (92.9%) | 44 (15.1%)  248 (84.9%) | 0.67 | 0.05 | 0.41 | 1.00 |
| Face-to-face 1:1 session (one off)  Yes  No | 4 (28.6%)  10 (71.4%) | 72 (24.7%)  220 (75.3%) | 0.11 | 0.02 | 0.74 | 1.00 |
| Online 1:1 session (one off)  Yes  No | 4 (28.6%)  10 (71.4%) | 72 (24.7%)  220 (75.3%) | 0.11 | 0.02 | 0.74 | 1.00 |
| Phone support  Yes  No | 9 (64.3%)  5 (35.7%) | 146 (50.0%)  146 (50.0%) | 1.09 | 0.06 | 0.30 | 1.00 |
| Written material (e.g., pamphlets)  Yes  No | 3 (21.4%)  11 (78.6%) | 62 (21.2%)  230 (78.8%) | <0.01 | 0.00 | 0.99 | 0.99 |
| Online material (e.g., videos, photos, etc.)  Yes  No | 3 (21.4%)  11 (78.6%) | 82 (28.1%)  210 (71.9%) | 0.30 | 0.03 | 0.59 | 1.00 |
| Motivational text messages  Yes  No | 3 (21.4%)  11 (78.6%) | 78 (26.7%)  214 (73.3%) | 0.19 | 0.03 | 0.66 | 1.00 |
| Self-guided online program  Yes  No | 1 (7.1%)  13 (92.9%) | 83 (28.4%)  209 (71.6%) | 3.04 | 0.10 | 0.08 | 0.96 |

Note: This table shows results of all subgroup analyses for Aboriginal and/or Torres Strait Islander status, comparing differences between Aboriginal and/or Torres Strait Islander peoples and non-Aboriginal and/or Torres Strait Islander people in responses, using Chi square tests. For all subgroup analyses, the Holm-Bonferroni correction was applied to adjust for multiple comparisons.

Additional file 4: Survey responses - Subgroup analyses by LGBTIQ+

|  | LGBTIQ+ | |  |  |  |  |
| --- | --- | --- | --- | --- | --- | --- |
|  | Yes | No | χ^2^ | Effect size | P value | Adjusted p value |
| **While on the waiting list, how interested would you have been to be supported to do an exercise program?** |  |  | 2.33 | 0.09 | 0.68 | 1.00 |
| Very Interested | 12 (26.1%) | 78 (30.2%) |  |  |  |  |
| Somewhat Interested | 15 (32.6%) | 75 (29.1%) |  |  |  |  |
| Not Sure | 9 (19.6%) | 66 (25.6%) |  |  |  |  |
| Somewhat Uninterested | 4 (8.7%) | 19 (7.4%) |  |  |  |  |
| Very Uninterested | 6 (13.0%) | 20 (7.8%0 |  |  |  |  |
| **How would you have liked to receive this exercise program(s)? (Tick all that apply)**  Face-to-face group session (one off)  Yes  No | 6 (13.0%)  40 (87.0%) | 29 (11.2%)  230 (88.8%) | 0.13 | 0.02 | 0.72 | 1.00 |
| Face-to-face group sessions (weekly)  Yes  No | 21 (45.7%)  25 (54.3%) | 91 (35.1%)  168 (64.9%) | 1.86 | 0.08 | 0.17 | 1.00 |
| Online group session (one off)  Yes  No | 3 (6.5%)  43 (93.5%) | 13 (5.0%)  246 (95.0%) | 0.18 | 0.02 | 0.67 | 1.00 |
| Online group sessions (weekly)  Yes  No | 8 (17.4%)  38 (82.6%) | 34 (13.1%)  225 (86.9%) | 0.60 | 0.04 | 0.44 | 1.00 |
| Face-to-face individual session (one off)  Yes  No | 20 (43.5%)  26 (56.5%) | 53 (20.5%)  206 (79.5%) | 11.37 | 0.20 | **<0.01*** | 1.00 |
| Online individual session (one off)  Yes  No | 8 (17.4%)  38 (82.6%) | 22 (8.5%)  237 (91.5%) | 3.49 | 0.11 | 0.06 | 0.66 |
| Phone support  Yes  No | 11 (23.9%)  35 (76.1%) | 63 (24.3%)  196 (75.7%) | 0.00 | 0.00 | 0.95 | 0.95 |
| Written material (e.g., pamphlets)  Yes  No | 10 (21.7%)  36 (78.3%) | 39 (15.1%)  220 (84.9%) | 1.29 | 0.07 | 0.26 | 1.00 |
| Online material (e.g., videos, photos, etc.)  Yes  No | 14 (30.4%)  32 (69.6%) | 63 (24.3%)  196 (75.7%) | 0.77 | 0.05 | 0.38 | 1.00 |
| Motivational text messages  Yes  No | 13 (28.3%)  33 (71.7%) | 59 (22.8%)  200 (77.2%) | 0.65 | 0.05 | 0.42 | 1.00 |
| Self-guided online program  Yes  No | 15 (32.6%)  31 (67.4%) | 69 (26.6%)  190 (73.4%) | 0.70 | 0.05 | 0.40 | 1.00 |
| **What type of exercise programs would you have been interested in? (Tick all that apply)**  Walking  Yes  No | 28 (60.9%)  18 (39.1%) | 164 (63.3%)  95 (36.7%) | 0.10 | 0.02 | 0.75 | 1.00 |
| Jogging  Yes  No | 8 (17.4%)  38 (82.6%) | 21 (8.1%)  238 (91.9%) | 3.91 | 0.11 | 0.05 | 0.25 |
| Interval Training (e.g., CrossFit)  Yes  No | 7 (15.2%)  39 (84.8%) | 30 (11.6%)  229 (88.4%) | 0.48 | 0.04 | 0.49 | 1.00 |
| Cycling  Yes  No | 10 (21.7%)  36 (78.3%) | 25 (9.7%)  234 (90.3%) | 5.62 | 0.14 | 0.02 | 0.14 |
| Weights (e.g., Resistance Training, Gym)  Yes  No | 19 (41.3%)  27 (58.7%) | 85 (32.8%)  174 (67.2%) | 1.25 | 0.06 | 0.26 | 1.00 |
| Swimming / Aquatic exercise  Yes  No | 26 (56.5%)  20 (43.5%) | 78 (30.1%)  181 (69.9%) | 12.12 | 0.20 | **<0.01*** | **<0.01*** |
| Yoga/Pilates  Yes  No | 25 (54.3%)  21 (45.7%) | 98 (37.8%)  161 (62.2%) | 4.43 | 0.12 | 0.04 | 0.24 |
| Circuits  Yes  No | 4 (8.7%)  42 (91.3%) | 22 (8.5%)  237 (91.5%) | 0.00 | 0.00 | 0.96 | 0.96 |
| Dancing  Yes  No | 20 (43.5%)  26 (56.5%) | 46 (17.8%)  213 (82.2%) | 15.24 | 0.23 | **<0.01*** | **<0.01*** |
| **While on the waiting list, how interested would you have been in being supported to complete a Healthy Eating program?**  Very Interested | 13 (28.3%) | 94 (36.3%) | 3.36 | 0.11 | 0.65 | 1.00 |
| Somewhat Interested | 18 (39.1%) | 73 (28.2%) |  |  |  |  |
| Not Sure | 5 (10.9%) | 42 (16.2%) |  |  |  |  |
| Somewhat Uninterested | 6 (13.0%) | 30 (11.6%) |  |  |  |  |
| Very Uninterested | 4 (8.7%) | 19 (7.3%) |  |  |  |  |
| Missing | 0 (0.0%) | 1 (0.4%) |  |  |  |  |
| **How would you have liked to receive this Healthy Eating program? (Tick all that apply)**  Face-to-face group session (one off)  Yes  No | 8 (17.4%)  38 (82.6%) | 20 (7.7%)  239 (92.3%) | 4.38 | 0.12 | 0.04 | 0.44 |
| Face-to-face group sessions (weekly)  Yes  No | 17 (37.0%)  29 (63.0%) | 53 (20.5%)  206 (79.5%) | 6.01 | 0.14 | 0.01 | 0.12 |
| Online group session (one off)  Yes  No | 6 (13.0%)  40 (87.0%) | 19 7.3%)  240 92.7%) | 1.69 | 0.08 | 0.19 | 0.95 |
| Online group sessions (weekly)  Yes  No | 7 (15.2%)  39 (84.8%) | 42 (16.2%)  217 (83.8%) | 0.03 | 0.01 | 0.87 | 0.87 |
| Face-to-face individual session (one off)  Yes  No | 15 (32.6%)  31 (67.4%) | 51 (19.7%)  208 (80.3%) | 3.84 | 0.11 | 0.05 | 0.50 |
| Online individual session (one off)  Yes  No | 15 (32.6%)  31 (67.4%) | 25 (9.7%)  234 (90.3%) | 18.07 | 0.25 | **<0.01*** | **<0.01*** |
| Phone support  Yes  No | 10 (21.7%)  36 (78.3%) | 60 (23.2%)  199 (76.8%) | 0.05 | 0.01 | 0.83 | 1.00 |
| Written material (e.g., pamphlets)  Yes  No | 15 (32.6%)  31 (67.4%) | 77 (29.7%)  182 (70.3%) | 0.15 | 0.02 | 0.70 | 1.00 |
| Online material (e.g., videos, photos, etc.)  Yes  No | 20 (43.5%)  26 (56.5%) | 77 (29.7%)  182 (70.3%) | 3.40 | 0.11 | 0.07 | 0.63 |
| Motivational text messages  Yes  No | 12 (26.1%)  34 (73.9%) | 45 (17.4%)  214 (82.6%) | 1.95 | 0.08 | 0.16 | 1.00 |
| Cooking workshops  Yes  No | 25 (54.3%)  21 (45.7%) | 87 (33.6%)  172 (66.4%) | 7.24 | 0.16 | 0.01 | 0.12 |
| Supermarket tours  Yes  No | 7 (15.2%)  39 (84.8%) | 23 (8.9%)  236 (91.1%) | 1.77 | 0.08 | 0.18 | 1.00 |
| Self-guided online program  Yes  No | 17 (37.0%)  29 (63.0%) | 67 (25.9%)  192 (74.1%) | 2.41 | 0.09 | 0.12 | 0.96 |
| **While on the waiting list, how interested would you have been to be supported to do a Sleep Education program?**  Very Interested | 17 (37.0%) | 80 (30.9%) | 4.66 | 0.13 | 0.46 | 1.00 |
| Somewhat Interested | 14 (30.4%) | 71 (27.4%) |  |  |  |  |
| Not Sure | 4 (8.7%) | 57 (22.0%) |  |  |  |  |
| Somewhat Uninterested | 6 (13.0%) | 26 (10.0%) |  |  |  |  |
| Very Uninterested | 5 (10.9%) | 24 (9.3%) |  |  |  |  |
| Missing | 0 (0.0%) | 1 (0.4%) |  |  |  |  |
| **How would you have liked to receive this Sleep Education program? (Tick all that apply)**  Face-to-face group session (one off)  Yes  No | 7 (15.2%)  39 (84.8%) | 18 (6.9%)  241 (93.1%) | 3.55 | 0.11 | 0.06 | 0.48 |
| Face-to-face group sessions (weekly)  Yes  No | 9 (19.6%)  37 (80.4%) | 43 (16.6%)  216 (83.4%) | 0.24 | 0.03 | 0.62 | 1.00 |
| Online group session (one off)  Yes  No | 5 (10.9%)  41 (89.1%) | 19 (7.3%)  240 (92.7%) | 0.67 | 0.05 | 0.41 | 1.00 |
| Online group sessions (weekly)  Yes  No | 7 (15.2%)  39 (84.8%) | 32 (12.4%)  227 (87.6%) | 0.29 | 0.03 | 0.59 | 1.00 |
| Face-to-face 1:1 session (one off)  Yes  No | 18 (39.1%)  28 (60.9%) | 55 (21.2%)  204 (78.8%) | 6.87 | 0.15 | 0.01 | 0.10 |
| Online 1:1 session (one off)  Yes  No | 16 (34.8%)  30 (65.2%) | 35 (13.5%)  224 (86.5%) | 12.69 | 0.21 | **<0.01*** | **<0.01*** |
| Phone support  Yes  No | 13 (28.3%)  33 (71.7%) | 63 (24.3%)  196 (75.7%) | 0.32 | 0.03 | 0.57 | 1.00 |
| Written material (e.g., pamphlets)  Yes  No | 14 (30.4%)  32 (69.6%) | 68 (26.3%)  191 (73.7%) | 0.35 | 0.03 | 0.56 | 1.00 |
| Online material (e.g., videos, photos, etc.)  Yes  No | 21 (45.7%)  25 (54.3%) | 75 (29.0%)  184 (71.0%) | 5.05 | 0.13 | 0.03 | 0.27 |
| Motivational text messages  Yes  No | 9 (19.6%)  37 (80.4%) | 50 (19.3%)  209 (80.7%) | 0.00 | 0.00 | 0.97 | 0.97 |
| Self-guided online program  Yes  No | 24 (52.2%)  22 (47.8%) | 71 (27.4%)  188 (72.6%) | 11.17 | 0.19 | **<0.01*** | **<0.01*** |
| **While on the waiting list, how interested would you have been to be supported to do receive** **additional mental health support?**  Very Interested | 29 (63.0%) | 137 (52.9%) | 3.98 | 0.12 | 0.41 | 1.00 |
| Somewhat Interested | 12 (26.1%) | 78 (30.1%) |  |  |  |  |
| Not Sure | 2 (4.3%) | 33 (12.7%) |  |  |  |  |
| Somewhat Uninterested | 2 (4.3%) | 6 (2.3%) |  |  |  |  |
| Very Uninterested | 1 (2.2%) | 5 (1.9%) |  |  |  |  |
| **How would you have liked to receive this Mental Health support program? (Tick all that apply)**  Face-to-face group session (one off)  Yes  No | 8 (17.4%)  38 (82.6%) | 21 (8.1%)  238 (91.9%) | 3.91 | 0.11 | 0.05 | 0.45 |
| Face-to-face group sessions (weekly)  Yes  No | 19 (41.3%)  27 (58.7%) | 64 (24.7%)  195 (75.3%) | 5.43 | 0.13 | 0.02 | 0.22 |
| Online group session (one off)  Yes  No | 4 (8.7%)  42 (91.3%) | 13 (5.0%)  246 (95.0%) | 1.00 | 0.06 | 0.32 | 1.00 |
| Online group sessions (weekly)  Yes  No | 7 (15.2%)  39 (84.8%) | 38 (14.7%)  221 (85.3%) | 0.01 | 0.01 | 0.92 | 0.92 |
| Face-to-face 1:1 session (one off)  Yes  No | 15 (32.6%)  31 (67.4%) | 61 (23.6%)  198 (76.4%) | 1.71 | 0.08 | 0.19 | 1.00 |
| Online 1:1 session (one off)  Yes  No | 17 (37.0%)  29 (63.0%) | 59 (22.8%)  200 (77.2%) | 4.20 | 0.12 | 0.04 | 0.40 |
| Phone support  Yes  No | 26 (56.5%)  20 (43.5%) | 128 (49.4%)  131 (50.6%) | 0.79 | 0.05 | 0.38 | 1.00 |
| Written material (e.g., pamphlets)  Yes  No | 9 (19.6%)  37 (80.4%) | 56 (21.6%)  203 (78.4%) | 0.10 | 0.02 | 0.75 | 1.00 |
| Online material (e.g., videos, photos, etc.)  Yes  No | 17 (37.0%)  29 (63.0%) | 69 (26.6%)  190 (73.4%) | 2.05 | 0.08 | 0.15 | 1.00 |
| Motivational text messages  Yes  No | 19 (41.3%)  27 (58.7%) | 61 (23.6%)  198 (76.4%) | 6.36 | 0.15 | 0.01 | 0.12 |
| Self-guided online program  Yes  No | 17 (37.0%)  29 (63.0%) | 66 (25.5%)  193 (74.5%) | 2.60 | 0.09 | 0.11 | 0.88 |

Note: This table shows results of all subgroup analyses for LGBTIQ+ status, comparing differences between LGBTIQ+ and non-LGBTIQ+ in responses, using Chi square tests. For all subgroup analyses, the Holm-Bonferroni correction was applied to adjust for multiple comparisons.
